# Preparation, Characterization and Pharmacokinetic Study of Xiangfu Siwu Decoction Essential Oil/β-Cyclodextrin Inclusion Complex

**DOI:** 10.3390/molecules200610705

**Published:** 2015-06-10

**Authors:** Junzuan Xi, Dawei Qian, Jinao Duan, Pei Liu, Zhenhua Zhu, Jianming Guo, Yang Zhang, Ying Pan

**Affiliations:** 1 Jiangsu Key Laboratory for High Technology Research of Traditional Chinese Medicine Formulae, Nanjing University of Chinese Medicine, Nanjing 210023, China; E-Mails: xjzd666@163.com(J.X.); liupei@njutcm.edu.cn (P.L.); 04040416@163.com (Z.Z.); njuguo@njutcm.edu.cn (J.G.); zy91923@163.com (Y.Z.); ptianxie@126.com (Y.P.); 2 Jiangsu Collaborative Innovation Center of Chinese Medicinal Resources Industrialization, Nanjing University of Chinese Medicine, Nanjing 210023, China

**Keywords:** Xiangfu Siwu decoction, essential oil, β-cyclodextrin, inclusion complex, pharmacokinetics

## Abstract

Xiang-Fu-Si-Wu Decoction (XFSWD), a famous Chinese herbal formula, is considered an effective prescription for treating primary dysmenorrhea. The essential oil is a significant effective ingredient of XFSWD. However, its volatility, instability and poor water-solubility influence its pharmacodynamic effects. β-Cyclodextrin (β-CD) has the intrinsic ability to form specific inclusion complexes with such drugs to enhance their stability, solubility and bioavailability. The aim of this study was thus to compare the pharmacokinetic characteristics and the oral bioavailability of XFSWD essential oil (XEO) and its β-CD inclusion complex after oral administration to rats. A simple, rapid, and sensitive ultra-high performance liquid chromatography tandem mass spectrometry (UPLC-MS/MS) method was developed for the simultaneous quantification of five active components of XEO in rat plasma. The *in vivo* data showed that XEO/β-CD inclusion complex displayed higher maximum plasma concentration (*C*_max_), longer half-time (*T*_1/2_) and bigger area under the concentration-time curve (*AUC*_0–24 h_). These results demonstrated that the formation of β-CD inclusion complex has significantly increased the oral bioavailability of the drugs in rats than free oil.

## 1. Introduction

Traditional Chinese Medicine (TCM) has been proved to be effective and safe in clinical applications for thousands of years and it plays an increasingly important role in evidence-based personalized medicine, which is a new trend and a hot research topic in medical development [1]. Xiang-Fu-Si-Wu Decoction (XFSWD), a famous Chinese herbal formula, is considered an effective prescription for treating primary dysmenorrhea [2], which is reported as one of the most common gynecological disorders in young women [3,4]. This prescription, originally created by Lian-Fu Liang during the Qing dynasty, is composed of seven herbs: Rehmanniae Radix Praeparata, Angelicae sinensis Radix, Chuanxiong Rhizoma, Paeoniae Radix Alba, Cyperi Rhizoma, Aucklandiae Radix and Corydalis Rhizoma, in the ratio of 4:3:1.5:1.5:1.5:1:1.5 on a dry weight basis, respectively. Four herbs of XFSWD, which are Angelicae sinensis Radix, Chuanxiong Rhizoma, Cyperi Rhizoma and Aucklandiae Radix, are rich in essential oil. Our previous research indicated the close correlation between volatile components and bioactivity of the prescription [5].

However, XFSWD essential oil (XEO) is volatile, sensitive to light, oxygen, humidity and high temperature, it has a pungent smell, and is poorly water-soluble [6,7,8]. Among these properties, the volatility and instability may cause storage and quality control difficulties, the poor water-solubility may substantially decrease the bioavailability of the drug and hence limit its pharmacodynamic effect, and the pungent smell may result in poor oral administration compliance. Therefore, developing a pharmaceutic method to increase the stability and solubility of such drugs so as to improve their bioavailability is considered an important task in the pharmaceutical field [9].

Cyclodextrins (CD) are non-toxic macrocyclic oligosaccharides composed of 6–9 glucopyranose units (namely α-, β-, γ- or δ-CD, respectively) with a relatively hydrophilic surface and hydrophobic central cavity. Through host-guest interactions with the organic molecules, drug-CD inclusion complexes are formed so that the included drugs can be protected against hydrolysis, oxidation and photodecomposition. In recent years, β-cyclodextrin (β-CD) has been identified as an attractive material for drug inclusion, thanks to its low biotoxicity and high biocompatibility, which have allowed it to be successfully applied to improve the chemical stability, solubility and bioavailability of a number of poorly soluble compounds in oral drug delivery [10,11,12,13]. The same technology also can be used in TCM.

**Figure 1:**
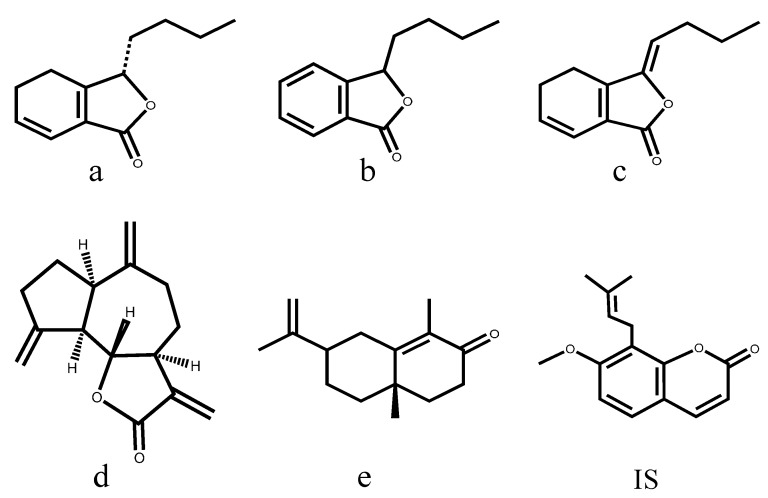
Chemical structures of senkyunolide A (**a**); 3-*n*-butylphthalide (**b**); *Z*-ligustilide (**c**); dehydrocostus lactone (**d**); α-cyperone (**e**) and osthole (**IS**).

In this present study, β-CD complexation was attempted for the process of pharmaceutical preparation of XEO. The XEO/β-CD inclusion complex was prepared by the coprecipitation method and characterized by different analytical techniques, including differential scanning calorimetry (DSC), Fourier Transform infrared spectroscopy (FT-IR), X-ray diffraction (XRD) and scanning electron microscope (SEM). The objective of this study was to investigate and compare the pharmacokinetic characteristics of XEO and its β-CD inclusion complex in rat plasma after oral administration. The components senkyunolide A, 3-*n*-butylphthalide, *Z*-ligustilide, dehydrocostus lactone and α-cyperone are the main bioactive ingredients in XEO. These components were selected indicators for evaluation in the pharmacokinetic study. Their chemical structures are shown in Figure 1.

Pharmacokinetic studies of some of these analytes such as senkyunolide A, dehydrocostus lactone and α-cyperone indicate they are poorly or not absorbed. It is essential to develop a sensitive and rapid ultra-high performance liquid chromatography tandem mass spectrometry (UPLC-MS/MS) method for the quantification of these five components in rat plasma. To the best of our knowledge, this is the first pharmacokinetic study on XEO and its β-CD inclusion complex by this method [14,15,16,17]. The pharmacokinetic parameters of both formulations were analysed by a non-compartmental model.

## 2. Results and Discussion

### 2.1. Characterization of EXO/β-CD Inclusion Complex

#### 2.1.1. DSC

**Figure 2:**
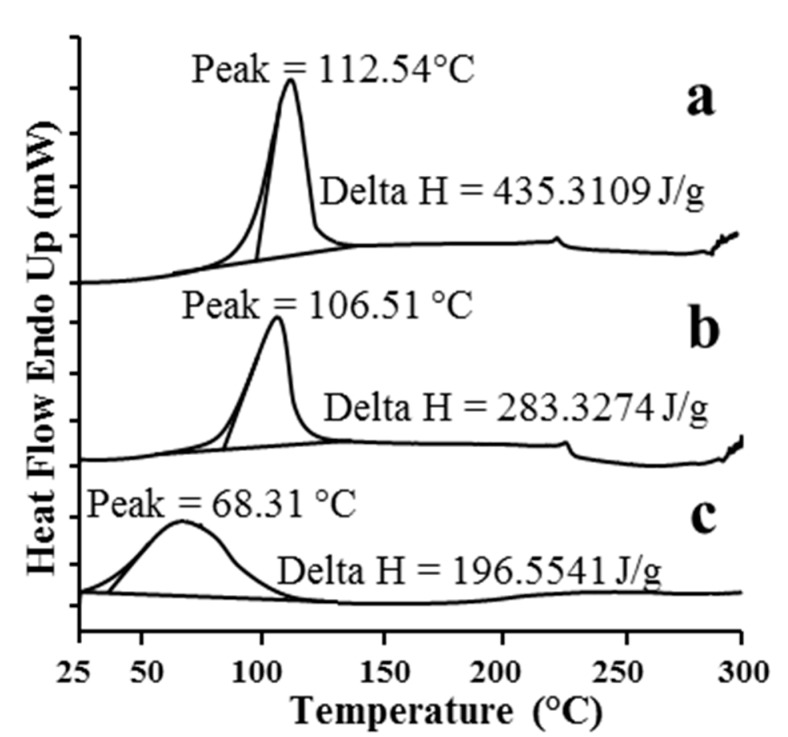
DSC grams of β-CD (**a**); the physical mixture (**b**) and the inclusion complex (**c**).

DSC is a powerful qualitative analytical technique for determining the thermal properties of solid cyclodextrin complexes [18]. This technique is widely used to investigate the changes in thermal behavior in inclusion complex preparation according to a standard procedure such as coprecipitation. In general, complexation results in the disappearance of endothermic peaks, appearance of new peaks, and peak broadening or shifting to different temperatures, which indicate a change in the crystal lattice, melting, boiling or sublimation points [19]. DSC thermograms of pure β-CD, the physical mixture and the inclusion complex are depicted in Figure 2. The thermograms of pure β-CD (Figure 2a) and the physical mixture (Figure 2b) show an endothermic peak at 112 °C and 106 °C, respectively, whereas in the thermogram of the inclusion complex (Figure 2c), the intensity of the broader endothermic peak was reduced and the peak position also shifted to 68 °C. This may indicate less or no interaction between the drug and β-CD in the physical mixture and confirm the formation of a host-guest inclusion complex.

#### 2.1.2. FT-IR

**Figure 3:**
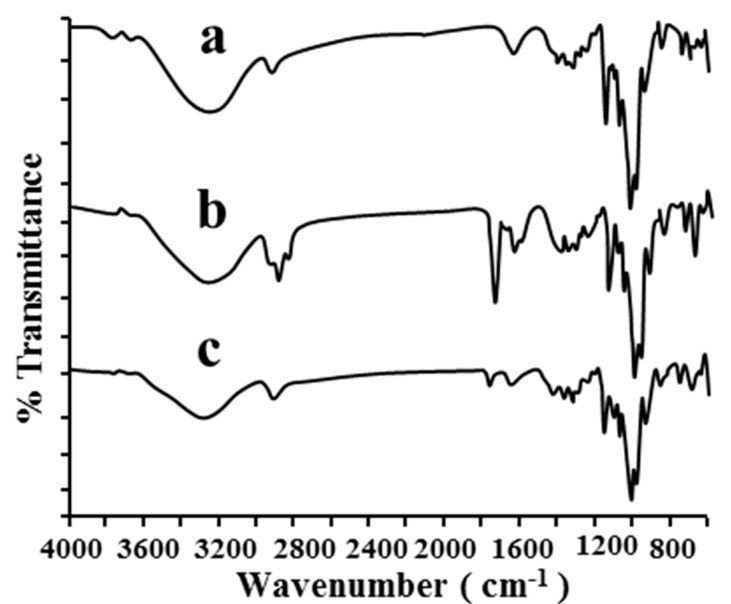
FT-IR spectra of β-CD (**a**); the physical mixture (**b**) and the inclusion complex (**c**).

The variation of the shape, shift, or intensity of IR absorption peaks of the guest or host can provide information about the occurrence of inclusion complex formation [20]. The FT-IR spectra of pure β-CD, the physical mixture and the inclusion complex are presented in Figure 3. Changes in the shape, position and intensity of the absorption bands of the different samples were observed. The physical mixture (Figure 3b) showed strong absorption peaks at 2935, 1767 and 710 cm^−1^, whereas there were obvious changes in the spectrum of the inclusion complex (Figure 3c), which was similar to that of pure β-CD (Figure 3a), showing disappearance or reduction of the absorption intensities of the corresponding bands. This result suggested that the functional groups of the drug were included within the apolar cavity of β-CD in the complex.

#### 2.1.3. XRD

**Figure 4:**
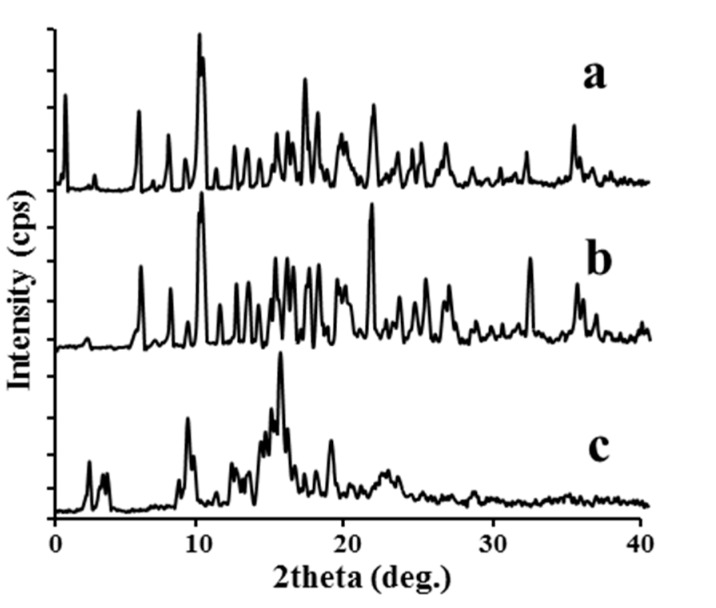
XRD patterns of β-CD (**a**); the physical mixture (**b**) and the inclusion complex (**c**).

XRD is a useful method for the detection of β-CD encapsulation and it has been used to assess the degree of crystallinity of a given sample [21]. The formation of a solid inclusion complex can be confirmed by the XRD pattern, because the crystallinity is generally shifted to a more amorphous or semi-crystalline structure in inclusion complexes [22]. Hence the complex usually exhibits less numbered as well as less intense peaks [23]. The XRD patterns of pure β-CD, the physical mixture and the inclusion complex were illustrated in Figure 4. The diffractogram of pure β-CD shown in Figure 4a displayed numerous characteristic peaks, which indicated its crystalline form [24]. The diffraction pattern of the physical mixture (Figure 4b) was just the superposition of the guest molecule and β-CD patterns. This may indicate that there was no interaction between the drug and β-CD in the physical mixture. In contrast, the absence of many peaks in the pattern of the inclusion complex (Figure 4c) indicated its amorphous or semi-crystalline state. This revealed a transformation that might be attributed to inclusion in the β-CD cavity.

#### 2.1.4. SEM

**Figure 5:**
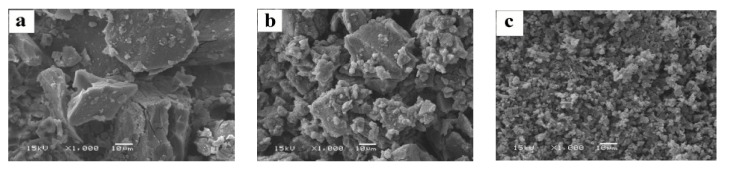
SEM images of β-CD (**a**); the physical mixture (**b**) and the inclusion complex (**c**).

SEM analysis is ideal for measuring the surface roughness and visualizing the surface texture of a substance [25]. The SEM images of pure β-CD, the physical mixture and the inclusion complex, which were utilized to evaluate the effect of coprecipitation process, are shown in Figure 5. A drastic change in morphology and change in crystalline nature was observed for the inclusion complex (Figure 5c), indicating that there was an apparent interaction between the drug and β-CD during the formation of the inclusion complex [26,27].

### 2.2. Liquid Chromatography and Mass Spectrometry Condition Optimization

For the purpose of achieving short retention times and symmetric peak shape, several combinations of acetonitrile, methanol, formic acid and acetic acid were applied to optimize the mobile phase. It was found that acetonitrile-water system provided good separation with a low baseline. Moreover, the use of 0.1% formic acid in the water phase helped in attaining a higher response and better peak sensitivity for the analytes. Hence acetonitrile-0.1% formic acid with gradient elution was employed and selected as the most suitable mobile phase to give appropriate retention times and low background noise. The ESI sources were set to both positive and negative ionization mode. The results showed that the analytes responded better in positive ion mode.

### 2.3. Method Validation

#### 2.3.1. Specificity and Selectivity

**Figure 6:**
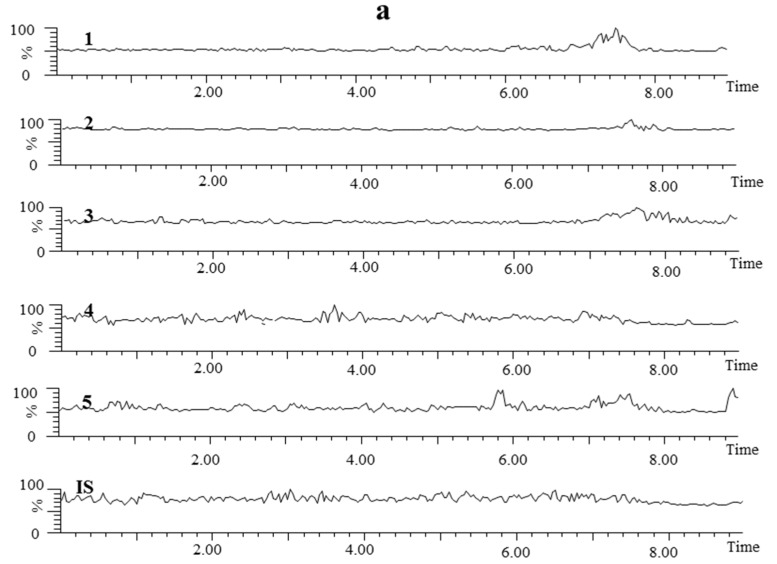

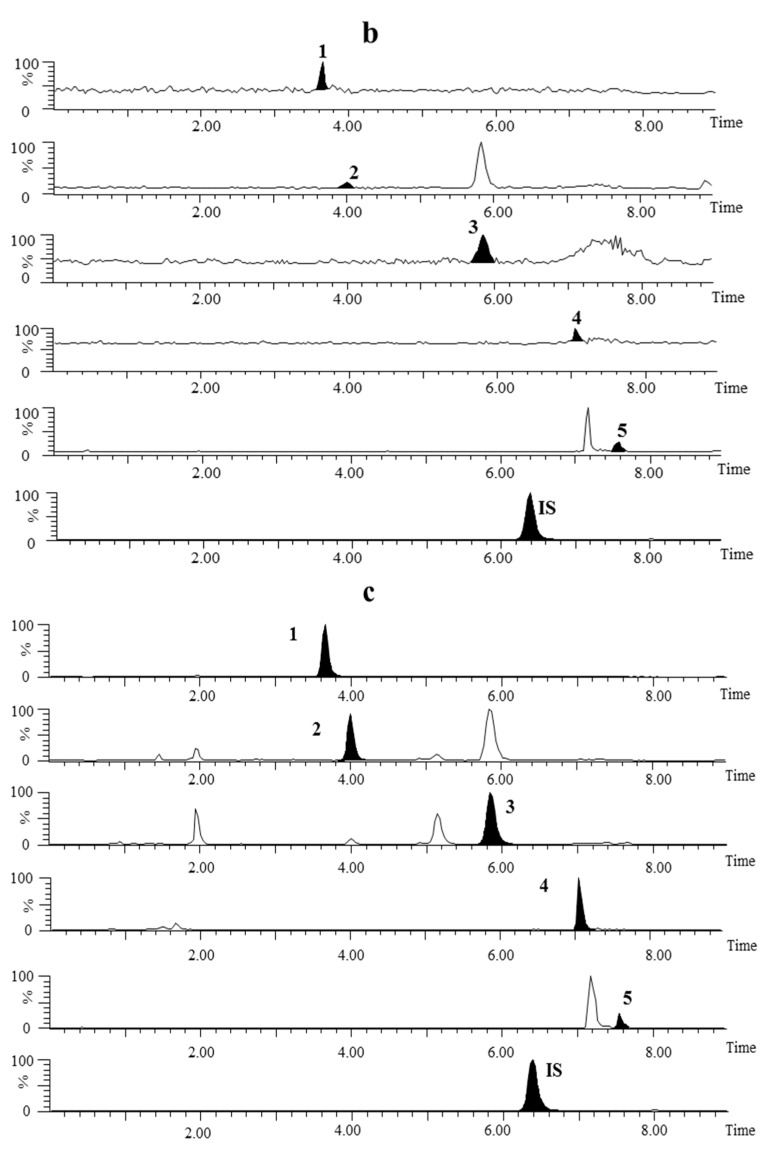
MRM chromatograms of each component in (**a**) blank plasma; (**b**) blank plasma spiked with a solution of standard mixture at the LLOQ; (**c**) plasma sample at 30 min after oral XEO/β-CD inclusion complex. Component ideitification: (1) senkyunolide A; (2) 3-*n*-butylphthalide; (3) *Z*-ligustilide; (4) dehydrocostus lactone; (5) α-cyperone; IS (osthole).

Typical MRM chromatograms of blank plasma, blank plasma spiked with a solution of standard mixture at the lower limit of quantitation (LLOQ) and a real rat plasma sample obtained at 30 min after oral administration of XEO/β-CD inclusion complex are shown in Figure 6. The retention times were about 3.62, 3.96, 5.80, 7.05, 7.56 and 6.38 min for senkyunolide A, 3-*n*-butylphthalide, *Z*-ligustilide, dehydrocostus lactone, α-cyperone and osthole, respectively. No endogenous substances in plasma interfered with the assay of the analytes and IS. In addition, 3-*n*-butylphthalide and *Z*-ligustilide are isomers. The big peak in Lane 2 of Figure 6b is *Z*-ligustilide. The retention time of α-cyperone reference substance is 7.56 min. Due to the plasma sample processing conditions, some α-cyperone was transformed into β-cyperone, which is the big peak in Lane 5 of Figure 6b.

#### 2.3.2. Linearity and LLOQ

Mean linear equations of the calibration curves (*n* = 6) were as follows: ***y*** = 1.1204***x*** − 0.0027 (***r*** = 0.9967, senkyunolide A), ***y*** = 0.0194***x*** − 0.0254 (***r*** = 0.9960, 3-*n*-butylphthalide), ***y*** = 0.3471***x*** + 0.0443 (***r*** = 0.9986, *Z*-ligustilide), ***y*** = 0.0840***x*** + 0.0021 (***r*** = 0.9949, dehydrocostus lactone) and ***y*** = 0.4084***x*** + 0.0068 (***r*** = 0.9971, α-cyperone). The lowest concentrations with RSD < 20% were taken as LLOQs and were found to be 4.6 ng/mL for senkyunolide A, 61.7 ng/mL for 3-*n*-butylphthalide, 4.5 ng/mL for *Z*-ligustilide, 6.1 ng/mL for dehydrocostus lactone and 8.2 ng/mL for α-cyperone, respectively. The ranges were all sufficient for pharmacokinetic studies.

#### 2.3.3. Accuracy and Precision

The accuracy data in the present study ranged from 87% to 103% and the intra- and inter-day of precision were 3.0%–7.9% and 3.2%–9.4%, respectively. All the assay values were found to be within the accepted variable limits, indicating that the established method was accurate and precise.

#### 2.3.4. Extraction Recovery and Matrix Effect

The mean recoveries of the analytes and IS were within 65%–76% (RSD < 10%), and the corresponding matrix effects ranged from 89% to 96% (RSD < 10%), which manifested that ethyl acetate was a feasible and appropriate medium for the analytes and IS extraction, and moreover, there was no measurable matrix effect on the ionization of analytes and IS.

#### 2.3.5. Stability

The stability of QC samples under different conditions was evaluated based on peak areas in comparison with freshly prepared QC samples. The results indicated that these analytes were all stable with accuracy in the range from 86.4% to 97.6%.

### 2.4. Pharmacokinetics

**Figure 7:**
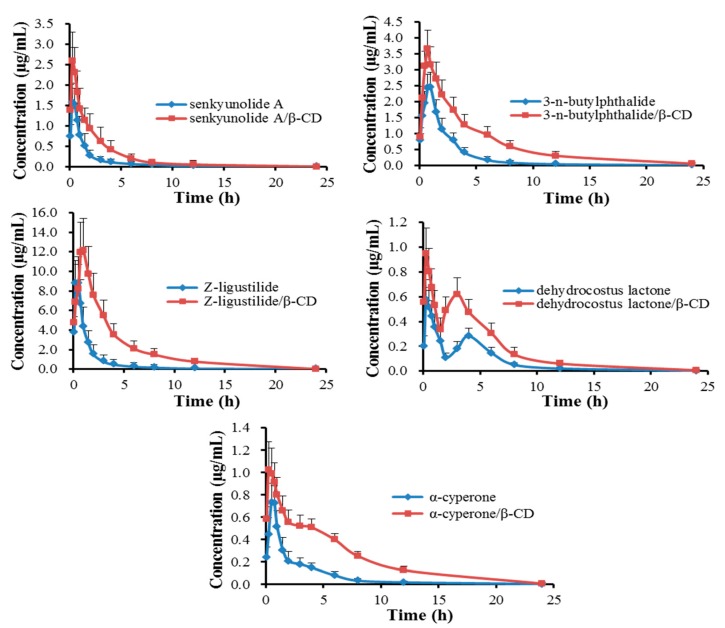
Mean (±S.D.) plasma concentration-time profiles of five active compounds after oral administration of XEO and its β-CD inclusion complex (*n* = 6).

The validated UPLC-MS/MS method was successfully applied for analysis of the plasma samples. Mean plasma concentration *vs.* time profiles of XEO and its β-CD inclusion complex are illustrated in Figure 7. Pharmacokinetic parameters were summarized in Table 1. The statistical results indicated that there was a significant difference between XEO and its β-CD inclusion complex.

**Table 1:** Summary of pharmacokinetic parameters (*n* = 6).

Components	Groups	Dose (mg/kg)	*C*_max_ (μg/mL)	*T*_max_ (h)	*T*_1/2_ (h)	MRT (h)	*AUC*_0–24 h_ (μg·h/mL)
Senkyunolide A	oil	13.14	1.59 ± 0.21	0.33 ± 0.13	3.73 ± 0.25	2.76 ± 0.52	2.40 ± 0.36
	oil/β-CD	13.41	2.60 ± 0.47	0.25 ± 0.00	4.43 ± 0.39	3.27 ± 0.25	6.08 ± 1.36
3-*n*-Butylphthalide	oil	34.66	2.48 ± 0.36	0.92 ± 0.13	4.05 ± 0.38	4.26 ± 0.28	6.51 ± 0.89
	oil/β-CD	35.38	3.69 ± 0.25	0.71 ± 0.10	4.81 ± 0.35	5.02 ± 0.60	16.68 ± 2.96
*Z*-Ligustilide	oil	116.08	8.80 ± 0.74	0.38 ± 0.14	3.21 ± 0.54	2.01 ± 0.12	13.29 ± 3.03
	oil/β-CD	84.75	11.43 ± 1.90	0.88 ± 0.14	4.16 ± 0.52	3.98 ± 0.25	38.69 ± 7.30
Dehydrocostus lactone	oil	3.64	0.58 ± 0.12	0.25 ± 0.00	3.81 ± 0.88	4.33 ± 0.30	1.92 ± 0.44
	oil/β-CD	5.73	0.87 ± 0.26	0.25 ± 0.00	4.20 ± 0.37	4.82 ± 0.16	4.33 ± 0.42
α-Cyperone	oil	8.18	0.75 ± 0.18	0.62 ± 0.14	3.05 ± 0.90	3.40 ± 0.52	1.81 ± 0.54
	oil/β-CD	7.69	1.05 ± 0.21	0.38 ± 0.14	4.83 ± 0.62	5.35 ± 0.30	5.53 ± 0.95

On the basis of the biopharmaceutics classification system (BCS), volatile compounds belong to class 2, with low solubility and high permeability, which mainly undergo passive transport across plasma membranes [28,29]. The hydrophilic mucus layer and unstirred water layer of gastrointestinal membrane are the main obstacles for the absorption of this class of drugs [30]. By comparing the results of two formulations, the inclusion complex showed a significant increase of maximum plasma concentration (*C*_max_), half-time (*T*_1/2_), mean residence time (MRT) and area under the concentration-time curve (*AUC*_0–24 h_) but a decrease in the time to reach maximum plasma concentration (*T*_max_) of the components, except for *Z*-ligustilide and dehydrocostus lactone. The plasma concentration reached the maximum swiftly after administration on account of its high permeability. The longer *T*_1/2_ and MRT may be due to a dynamic balance between the inclusion complex and free drug [31]. With increasing plasma concentration, the release rates of included drugs were gradually slowed down.

The decrease of *T*_max_ and the enhancement of *C*_max_ and *AUC*_0–24 h_ in the inclusion complex form may be ascribed to an increase of dissolution and water-solubility in the hydrophilic mucus layer and unstirred water layer of the gastrointestinal membrane, which resulted in improved permeation [32] because the drugs were embedded into the β-CD cavity and β-CD has a hydrophilic surface [33].

Another possible cause was the improvement of stability in bodily fluids, especially for *Z*-ligustilide and α-cyperone. The *T*_max_ shift of *Z*-ligustilide and dehydrocostus lactone may be attributed to the ability of release from inclusion complex. Some of the above conclusions are just conjecture, which requires further experimental confirmation.

The results revealed that the relative oral bioavailability of the five active compounds in the inclusion complex increased significantly in rats compared to those in the free oil. Therefore, β-CD is a applicable vector to increase drug solubility and absorption, and the development of a XEO delivery system can greatly improve its bioavailability.

## 3. Experimental Section

### 3.1. Materials and Reagents

β-CD was purchased from Jiangsu Fengyuan Biotechnology Co., Ltd (Jiangsu, China). The reference standards of senkyunolide A, 3-*n*-butylphthalide, *Z*-ligustilide, dehydrocostus lactone, α-cyperone and osthole (internal standard) were purchased from the National Institute for the Control of Pharmaceutical and Biological Products (Beijing, China). Their chemical structures are shown in Figure 1. HPLC grade acetonitrile and methanol were obtained from Tedia (Fairfield, NJ, USA). Analytical grade formic acid was purchased from Merck (Darmstadt, Germany). The deionized water was purified by a Millipore water purification system (Millipore, Milford, MA, USA) and filtered with 0.22 μm membranes. All other reagents used were of analytical grade. *Rehmanniae Radix Praeparata*, *Angelicae sinensis Radix*, *Chuanxiong Rhizoma*, *Paeoniae Radix Alba*, *Cyperi Rhizoma*, *Aucklandiae Radix* and *Corydalis Rhizoma* were purchased from Anhui Fengyuan Tongling Chinese Herbal Medicine Co., Ltd (Anhui, China). The corresponding author authenticated all of the materials, and the herbal drugs were verified according to the Chinese Pharmacopeia (2010 edition). Voucher specimens were deposited at the Jiangsu Key Laboratory for High Technology Research of Traditional Chinese Medicine (TCM) Formulae.

### 3.2. Animals

Experimental procedures were carried out in accordance with the Guide for the Care and Use of Laboratory Animals, and before the animal experiments were carried out, the procedures were approved by the Laboratory Animal Center of Nanjing University of Chinese Medicine. Twelve female SD rats weighing 250 ± 20 g (Certificate No. SCXK2012-0001) were provided by Beijing Weitong Lihua Experimental Animal Technology Co., Ltd (Beijing, China).

### 3.3. Preparation of XEO and its β-CD Inclusion Complex

XEO was extracted by steam distillation in the lab according to the ratio of the prescription. The XEO/β-CD inclusion complex was prepared by a coprecipitation method. Briefly, β-CD (30.00 g) and water (500 mL) were added to a flask which was placed on a constant temperature magnetic stirring apparatus. After stirring for an hour at 40 °C, the β-CD saturated solution was obtained. XEO (3 mL, 2.62 g) was dropwise added to the saturated β-CD solution with continuous agitation for an hour at the same temperature and rate. Then the solution was cooled to room temperature with continuous agitation and maintained overnight at 4 °C. Finally, the cold precipitated XEO/β-CD inclusion complex was recovered by vacuum filtration. The precipitate was washed with diethyl ether to remove XEO which was absorbed on the surface of β-CD and then freeze dried until the weight remained constant. The dried powder (28.34 g) was stored in an airtight desiccator at room temperature. In order to characterize the inclusion complex, the physical mixture of β-CD and XEO was prepared by mixing β-CD and XEO at a ratio of 10:1 (*m*/*v*) in a glass mortar until a homogeneous mixture was obtained. The contents of five active compounds are presented in Table 2. It shows that the ratios of analytes in essential oil and inclusion complex are all about 10:1, which agrees with the ratio of preparation of the β-CD inclusion complex.

**Table 2:** The contents of the five active compounds (%): (**a**) senkyunolide A; (**b**) 3-*n*-butylphthalide; (**c**) *Z*-ligustilide; (**d**) dehydrocostus lactone; (**e**) α-cyperone.

Compound	a	b	c	d	e
XEO	3.28	8.66	29.0	0.909	2.04
β-CD inclusion complex	0.335	0.884	2.12	0.143	0.192

### 3.4. Characterization of XEO/β-CD Inclusion Complex

#### 3.4.1. Differential Scanning Calorimetry Study (DSC)

The DSC thermograms for pure β-CD, the physical mixture and the inclusion complex were measured with a Diamond DSC instrument (Perkin Elmer, MA, USA). Samples were accurately weighed and heated in a crimped aluminum pan at a rate of 10 °C/min from 25 to 350 °C under a nitrogen flow of 40 mL/min. An empty aluminum pan was used as the reference.

#### 3.4.2. Fourier Transform Infrared Spectral Analysis (FT-IR)

The FT-IR spectra of pure β-CD, the physical mixture and the inclusion complex were recorded from 4000 to 600 cm^−1^ on a Tensor 37 FT-IR spectrophotometer (Bruker Optics, Karlsruhe, Germany) with 32 scans at a resolution of 4 cm^−1^. β-CD, the physical mixture and the inclusion complex were respectively mixed with spectrograde KBr powder at a mass ratio of 1:100. Then they were ground and pressed to discs of 8 mm diameter. FT-IR spectra were analysed by the OPUS 6.0 spectroscopy software.

#### 3.4.3. Powder X-ray Diffraction Analysis (XRD)

The powder XRD patterns of pure β-CD, the physical mixture and the inclusion complex were recorded in 2θ range 0–40° using a D/max-2500/PC diffractometer (Rigaku Corporation, Tokyo, Japan). The measurement conditions were as follows: graphite-monochromated Cu Kα radiation; voltage, 40 kV; current, 100 mA; DS = SS = 1°, RS = 0.3 mm.

#### 3.4.4. Scanning Electron Microscopy (SEM) Image Analysis

The surface morphologies of pure β-CD, the physical mixture and the inclusion complex were examined using a JSM-5610LV SEM instrument (JEOL, Tokyo, Japan) at 15 kV. The samples were sputtered with a thin layer of gold to improve the electrical conductivity prior to imaging.

### 3.5. Preparation of Calibration Standards and Quality Control Samples

Stock solutions of senkyunolide A (0.470 mg/mL), 3-*n*-butylphthalide (0.395 mg/mL), *Z*-ligustilide (0.462 mg/mL), dehydrocostus lactone (0.318 mg/mL), α-cyperone (0.419 mg/mL) and osthole (0.295 mg/mL) were prepared in methanol. The stock solutions were further diluted with methanol to obtain a set of standard working mixture solutions with a concentration range of 4.6 × 10^−3^–4.70 μg/mL for senkyunolide A, 61.7 × 10^−3^–7.90 μg/mL for 3-*n*-butylphthalide, 4.5 × 10^−3^–18.48 μg/mL for *Z*-ligustilide, 6.1 × 10^−3^–1.55 μg/mL for dehydrocostus lactone, 8.2 × 10^−3^–2.10 μg/mL for α-cyperone, respectively. Internal standard (IS) solution was prepared by dilution of its stock solution to 1.18 μg/mL with methanol. All solutions were stored at −20 °C before analysis. The solutions of standard mixture, IS and ethyl acetate with blank plasma at a volume ratio of 200:10:600:200 μL were mixed. Quality control (QC) samples at low, middle and high concentrations were 0.018, 0.294, 4.70 μg/mL for senkyunolide A; 0.123, 0.984, 7.90 μg/mL for 3-*n*-butylphthalide; 0.036, 0.576, 9.24 μg/mL for *Z*-ligustilide; 0.024, 0.194, 1.55 μg/mL for dehydrocostus lactone; 0.033, 0.262, 2.10 μg/mL for α-cyperone, respectively.

### 3.6. UPLC-MS/MS Instrument and Conditions

The chromatographic analysis was performed on an Acquity UPLC system (Waters Corp., Milford, MA, USA), consisting of a binary pump solvent management system, an online degasser, and an autosampler. An Acquity UPLC BEH C_18_ column (100 mm × 2.1 mm, 1.7 μm) was employed and the column temperature was maintained at 35 °C. The mobile phase was composed of A (0.1% formic acid) and B (acetonitrile) using a gradient elution of 42%–43% B at 0–6 min, 43%–95% B at 6–7 min, 95%–95% B at 7–8 min and 95%–42% B at 8–9 min with a flow rate set at 0.4 mL/min. The autosampler was conditioned at 4 °C and the injection volume was 2 μL. The mass spectrometry detection was performed using a Xevo Triple Quadrupole MS (Waters Corp.) equipped with an electrospray ionization source (ESI). Analytes were quantified by multiple-reaction monitoring (MRM) mode employing the following precursor-to-product ion pairs: 193.0 → 136.8 for senkyunolide A, 190.9 → 116.9 for 3-*n*-butylphthalide, 191.0 → 90.8 for *Z*-ligustilide, 231.0 → 194.9 for dehydrocostus lactone, 219.0 → 110.9 for α-cyperone, and 245.0 → 188.9 for osthole. The parameters in the source were set as follows: capillary voltage 3.5 kV; source temperature 150 °C; desolvation gas flow 1000 L/h; desolvation temperature 550 °C; cone gas flow 50 L/h. Dwell time was automatically set. Data were collected and analysed by MassLynx (Waters Corp.).

### 3.7. Method Validation

#### 3.7.1. Plasma Samples Preparation

Plasma samples (200 μL) were added with IS working solution (10 μL) and ethyl acetate (600 μL) in a 2 mL glass centrifuge tube. After vortexing for 2 min, the mixtures were centrifuged at 13,000 rpm for 10 min at 4 °C. Then the supernatant was transferred to another tube and evaporated to dryness in a rotary evaporator at 25 °C rapidly. The residue was reconstituted in 200 μL methanol and then centrifuged again at 13,000 rpm for 10 min. The supernatant (100 μL) was transferred to a vial for UPLC-MS/MS analysis.

#### 3.7.2. Specificity

The specificity was evaluated by comparing chromatograms of blank plasma, blank plasma spiked with the solutions of standard mixture and IS, and plasma samples obtained from rats administrated by XEO and its inclusion complex.

#### 3.7.3. Linearity and LLOQ

The linearity of each calibration curve was determined by plotting the peak area ratio (***y***) of analytes to IS *vs.* the nominal concentration (***x***) of analytes with weighted (1/***x***) least square linear regression. The lower limits of quantitation (LLOQ) of the assay was defined as the lowest concentration on the standard curve that can be quantitated with accuracy within 20% bias of the nominal concentration and RSD not exceeding 20%.

#### 3.7.4. Accuracy and precision

The accuracy and the precision of the assay for intra-day and inter-day determinations were evaluated by the analysis of three concentration levels of QC samples (*n* = 6) on the same day and on three consecutive validation days. The accuracy was expressed as RE (%) within 85%–115% from the nominal values, and the precision as RSD (%) within ±15% except for LLOQ, where it should be within 80%–120% for accuracy and less than 20% of precision.

#### 3.7.5. Extraction Recovery and Matrix Effect

The recovery was determined in sets of six replicate QC samples at three different concentrations by measuring the amount of each compound recovered after extraction and calculated by comparing the peak areas of the extracted samples with that of the unextracted standard solutions (*n =* 6). Matrix effect was measured via comparison of the peak responses obtained from samples where the extracted matrix was spiked with standard solutions to those obtained from neat standard solutions at equivalent concentrations.

#### 3.7.6. Stability

The stabilities of analytes in rat plasma were investigated by using QC samples stored under different temperature conditions for different periods of time which likely to be encountered during sample storage and the analytical process. Three replicate QC samples were tested for pre-treatment, post-treatment, three freeze-thaw cycles and long-term stabilities, respectively. Pre-treatment stability was assessed by exposing QC samples at room temperature for 4 h. Post-treatment was evaluated by placing QC samples in the autosampler at 4 °C for 24 h. And for freeze-thaw cycle stability assessment, QC samples were repeatedly freezed and thawed for three cycles at −80 to 20 °C. Long-term stability was carried out via placing QC samples at −80 °C for 2 weeks.

### 3.8. Pharmacokinetic Study

The rats were housed under standard conditions (GLP) with free access to food and water for one month. Before administration, rats were on overnight fast with free access to water. Then, they were randomly and equally divided into two groups (each consisting of six rats). One group received XEO orally administrated at a dose of 0.4 g/kg and the other group received XEO/β-CD inclusion complex orally administrated at a dose of 4 g/kg. XEO and its β-CD inclusion complex were both diluted with water to achieve appropriate concentrations corresponding to each rat. Both groups were administered about 2 mL per rat. Blood samples (0.3 mL) were collected from the orbit venous plexus of rats under ether anesthesia into heparinized Eppendorf tubes at 0.0833, 0.25, 0.5, 0.75, 1, 1.5, 2, 3, 4, 6, 8, 12 and 24 h after administration. The plasma was separated through centrifugation at 13,000 rpm for 10 min at 4 °C and stored at −80 °C until analysis. The pharmacokinetic parameters of analytes were calculated by DAS 3.2 software using non-compartment model analysis. Their statistical comparisons were made by two-sample *t*-test (SPSS), and p value less than 0.05 was considered to be statistically significant.

## 4. Conclusions

In this study, an UPLC-MS/MS method for the simultaneous determination of senkyunolide A, 3-*n*-butylphthalide, *Z*-ligustilide, dehydrocostus lactone and α-cyperone in rat plasma was developed for the first time, which provided adequate recovery and matrix effect with good precision and accuracy. Compared with the previously reported methods, the present method employed a simple and rapid extraction procedure for sample preparation, and offered higher sensitivity. The method was successfully applied to a pharmacokinetic study of EXO in rats. These pharmacokinetic results provided useful information for the further research on pharmaceutical study of XFSWD.
